# A CT-Based Radiomics Ensemble Model (CRIPEM) for Preoperative Prediction of Pathological Upstaging in Clear Cell Renal Cell Carcinoma

**DOI:** 10.3390/cancers18101558

**Published:** 2026-05-11

**Authors:** Yangyang Xia, Yihao Zhao, Changdong Yue, Jing Qi, Shaojian Zhang, Wenqiang Qi, Junxian Li, Chaobin Zhao, Yang Zheng, Benkang Shi, Xuewen Jiang

**Affiliations:** 1 Department of Urology, Qilu Hospital of Shandong University, Jinan 250012, China; xyysdu92@163.com (Y.X.);; 2 Laiwu People’s Hospital of Jinan City, Jinan 271100, China

**Keywords:** radiomics, bioinformatics analysis, intratumoral characteristics, peritumoral characteristics, integrated model

## Abstract

Background: Pathological upstaging (PU) of clear cell renal cell carcinoma (ccRCC) from clinical cT1 to pT3 stage often requires conversion from partial to radical nephrectomy. Preoperative PU prediction lacks objective, precise methods, hindering surgical decision-making. Methods: We developed and validated a computed tomography-based radiomics ensemble learning model (CRIPEM) integrating intratumoral and peritumoral features to predict PU in cT1 ccRCC. We enrolled a multicenter cohort of 309 cT1 ccRCC patients from three institutions, divided into training (*n* = 170), internal validation (*n* = 73), and external validation (*n* = 66) cohorts. Bioinformatics analysis confirmed that peritumoral regions harbor critical predictive value for PU. Results: A total of 7336 radiomic features were extracted from intratumoral and peritumoral (1, 2, 3 mm) regions on preoperative CT images, with 50 robust features retained via a rigorous five-step selection process. CRIPEM, fusing optimal base learners (IT-MLP for intratumoral features, PT1-RF for 1-mm peritumoral features), achieved area under the curve values of 0.872, 0.807, and 0.826 in the training, internal, and external validation cohorts, respectively. Subgroup, calibration, and decision curve analyses confirmed its stability, superiority over single base learners, and significant clinical net benefits, with individualized cases verifying clinical applicability. Conclusions: CRIPEM is an objective, accurate, and robust tool for preoperative PU prediction in cT1 ccRCC, which can optimize surgical strategy selection and improve patient clinical management.

## Simple Summary

Clinical T1 clear cell renal cell carcinoma may be pathologically upstaged to T3 stage after surgery. It requires surgeons to switch from partial nephrectomy to radical nephrectomy. Currently, there is no objective and accurate tools for preoperative prediction of such upstaging. We developed a CT-based radiomics ensemble model named CRIPEM by integrating intratumoral and peritumoral imaging features. Based on a multicenter cohort of 309 patients, the model showed favorable and stable predictive performance in training, internal validation, and external validation cohorts. This model can help clinicians assess upstaging risk preoperatively and select more reasonable surgical strategies. And it will improve the clinical management of patients in the future.

## 1. Key Points

Problem: Accurate preoperative prediction of pathological upstaging in clinical T1 clear cell renal cell carcinoma lacks objective tools, impeding optimal surgical decision-making.

Findings: Our validated CT-based radiomics ensemble model achieves excellent pathological upstaging prediction in clinical T1 clear cell renal cell carcinoma across multicenter cohorts.

## 2. Clinical Relevance Statement

This tool optimizes surgical strategy selection and improves clinical management for patients with clinical T1 renal cell carcinoma.

## 3. Introduction

Renal cell carcinoma (RCC) accounts for 2–3% of all malignancies [1,2]. Initial clinical staging, predominantly via high-resolution computed tomography (CT), guides clinical decisions regarding surgery [3], radiotherapy [4], or immunotherapy [5,6]. For patients with clinical stage cT1 RCC, partial or radical nephrectomy is recommended based on tumor size [7]. However, some patients preoperatively classed as cT1 may postoperatively exhibit pathological upstaging (PU), displaying pT3 features such as major vein or perinephric fat invasion [8]. As the surgical standard shifts from partial to radical nephrectomy between cT1 and pT3, this discrepancy has major clinical implications. Thus, improving the preoperative prediction of PU is critical for optimizing surgical decisions. When the risk of PU is high, surgeons should consider performing radical nephrectomy rather than partial nephrectomy for patients with T1a-stage tumors. For those with a low PU risk, partial nephrectomy remains an appropriate surgical option.

Current research on PU in RCC has predominantly focused on identifying clinicopathological predictors and evaluating associated oncological outcomes [9,10]. Factors such as male sex, T1b clinical stage, older age, and larger tumor size are consistently associated with an increased probability of PU, although the prognostic implication of such upstaging remains debated [11,12]. Traditional methods for predicting PU, including specific urine tests (e.g., urinary N-acetyl-β-D-glucosaminidase/creatinine ratio) [13], visual assessment of tumor contour irregularity [14], and the M-Index [15], often lack the objectivity and precision required for consistent surgical decisions. Consequently, objective and reliable predictive tools are urgently required. Habitat-based radiomics analysis [16,17], a method that quantifies intratumoral heterogeneity by dividing tumors into biologically distinct subregions (habitats), shows substantial promise for fulfilling this clinical requirement.

Therefore, we constructed and evaluated a habitat-based radiomics model (the CT-based clear cell RCC (ccRCC) intratumoral and peritumoral radiomics-based ensemble learning model: CRIPEM) using a multicenter cohort of 312 patients. First, we compared the differential gene expression profiles between patients with and without PU. Second, we extracted 7336 radiomic features from the intratumoral or peritumoral regions of preoperative CT images. Third, we employed a rigorous machine learning workflow—incorporating least absolute shrinkage and selection operator (LASSO) and Pearson analysis for feature selection—to build the predictive model. CRIPEM showed excellent and consistent predictive performance across training, internal validation, and external validation cohorts. The proposed predictive model effectively delineates tumor heterogeneity using habitat-based radiomics to improve the preoperative assessment of PU risk and optimize surgical decision-making.

## 4. Methods

### 4.1. Clinical Cohort Establishment

**Figure 1:**
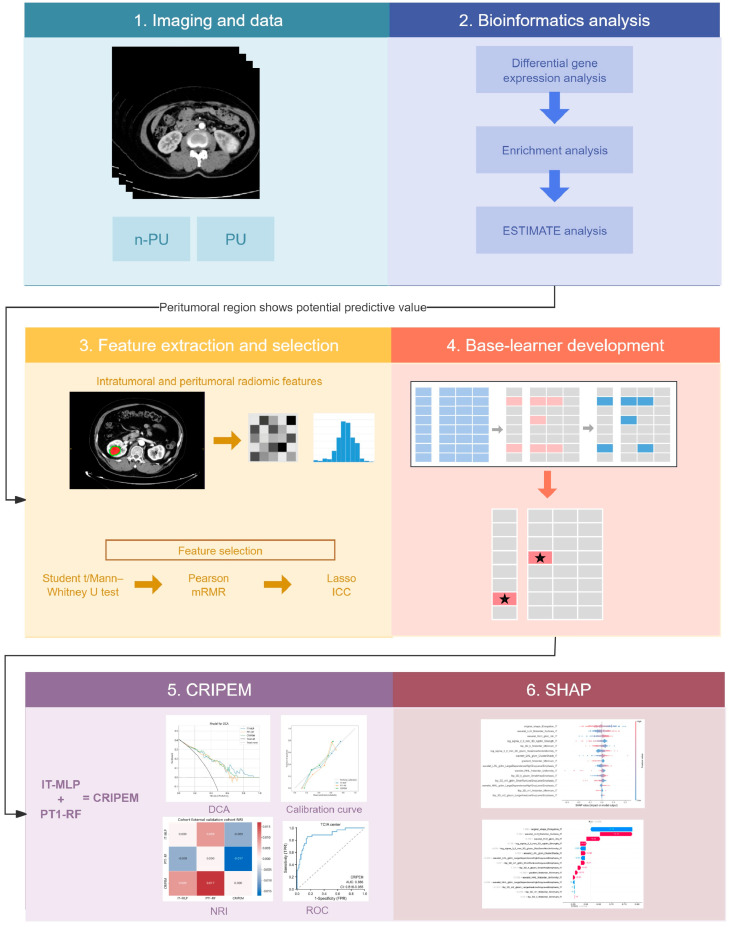
Schematic diagram of the study design and procedure.

This study was approved by the Institutional Ethics Committee (approval number: KYLL-2025SL-231; approval date: 17 October 2025) and conducted in accordance with the TRIPOD + AI statement and Declaration of Helsinki. The requirement for informed consent was waived owing to the retrospective nature of the study. We retrospectively collected CT images and clinical data of patients with ccRCC from three centers: Shandong University Qilu Hospital (QL), Shandong University Qilu Hospital (Qingdao) (QLQD), and The Cancer Imaging Archive (TCIA). After rigorous data review and quality control, 309 patients were included in the study. The inclusion and exclusion criteria are detailed in Supplementary Material SI. We merged patients from QL and TCIA to create a new cohort, which was randomly divided into training and internal validation cohorts at a ratio of 7:3. Patients from QLQD represented the external validation set. Clinical and pathological data (e.g., age, sex, and stage) were collected from electronic medical records and databases. The study enrollment process is illustrated in Figure S1. Figure 1 presents an overview of this study. For the external validation cohort from Qilu Hospital (Qingdao), we strictly applied the identical inclusion and exclusion criteria as the training and internal validation cohorts to ensure population consistency. Baseline characteristics (including age, sex, tumor laterality, and pathological stage) showed no statistically significant differences between the external validation cohort and the training/internal validation cohorts (all *p* > 0.05, Table 1), confirming the baseline comparability and clinical representativeness of this external cohort for cT1 ccRCC patients.

### 4.2. Bioinformatics Analysis

We obtained bulk RNA sequencing data for kidney ccRCC samples from The Cancer Genome Atlas database and matched them with imaging data from TCIA. The matched samples were then divided into PU and non-PU groups, and differential expression analysis was performed. Based on the identified differentially expressed genes, we conducted gene ontology functional annotation and gene set enrichment analysis to identify potential biological mechanisms. Additionally, we used the ESTIMATE algorithm to calculate and compare the stromal scores between groups to evaluate differences in the tumor microenvironment during PU.

### 4.3. CT Image Acquisition and Region-of-Interest Delineation

CT images were saved in DICOM format, and the renal cortex phase was selected for subsequent analysis. The CT scanning parameters for QL and QLQD are listed in Table S1. Two urologists used ITK-SNAP (version 4.4) to manually delineate the tumor region on each slice, which was confirmed by a urologist with over 15 years of experience. In each slice, the region of interest was expanded by image expansion to form a ring—shaped area with widths of 1, 2, and 3 mm, thereby obtaining different widths of the tumor margin. The three—dimensional segmentation results were saved as an NIfTI-format mask file. To evaluate intra-observer reproducibility, the same two physicians re-delineated the region of interest on 30 randomly selected patients one month later, determining the tumor margin using the same method. The results of the two measurements were used to calculate the intraclass correlation coefficient. The detailed CT scanning parameters of the primary center (QL) and external validation center (QLQD) are summarized in Table S1. Minor variability in scanning parameters (including tube current and reconstruction kernel) between the two centers was noted. To mitigate the domain shift caused by imaging protocol heterogeneity and improve the generalizability of radiomic features, we performed isotropic voxel resampling, Z-score standardization on all features prior to modeling, and only retained features with high intra-observer reproducibility (ICC > 0.8) via rigorous screening. These preprocessing steps minimized the impact of scanning protocol differences on feature stability.

### 4.4. Extraction of Imaging Features

Imaging features were extracted using the PyRadiomics software package v3.0.1, in accordance with Imaging Biomarker Standardization Initiative guidelines. The extracted features included first-order statistics, shape features, and texture features (based on GLCM, GLRLM, GLSZM, GLDM, and NGTDM). Further information on feature extraction is provided in Supplementary Material SII.

### 4.5. Feature Selection

A five-step feature selection strategy was adopted to select the most promising tumor- and peritumor-related radiomic features for diagnosis in the training cohort.

(1) Z-score standardization, followed by Student’s *t*-test and the Mann–Whitney U-test (retaining features with *p* < 0.05).
(2) Pearson’s correlation analysis to assess collinearity (retaining features with correlation coefficient > 0.9).
(3) Maximum relevance minimum redundancy algorithm to further optimize modeling features.
(4) LASSO algorithm to select features with non-zero coefficients for subsequent modeling.
(5) Intraclass correlation coefficient to evaluate feature repeatability and stability (retaining features with correlation coefficient > 0.8).

### 4.6. Development of Base Learners and Integrated Learning Models

The selected features were incorporated into eight common machine learning algorithms: support vector machine, logistic regression, k-nearest neighbors, random forest, extra trees, light gradient boosting machine, multilayer perceptron, and adaptive boosting. We established a set of criteria based on actual clinical needs to select the best base learners for each specific diagnosis. We then constructed the CRIPEM using an ensemble learning strategy; the detailed construction process is described in Supplementary Material SIII. The performance and robustness of CRIPEM were evaluated using a joint calibration curve, decision curve analysis, subgroup analysis, the area under the receiver operating characteristic curve (95% confidence interval), accuracy, sensitivity, specificity, positive predictive value, negative predictive value, and F1 score. Performance differences between the models were compared using the net reclassification improvement index. Parameter choices and feature selection are repeated independently within each training split.

### 4.7. Statistical Analysis

All data analyses and visualizations were performed using Python 3.7.12, R 4.3.3, and GraphPad Prism (version 9.5.1). Continuous variables were expressed as the mean ± standard deviation or median (interquartile range), whereas categorical variables were expressed as the frequency (percentage). Support vector machine, random forest, and extra trees algorithms were implemented using scikit-learn 1.0.2, whereas the light gradient boosting machine algorithm was implemented using the lightgbm 3.3.2 package. Statistical tests and modeling were performed using Statistical Models 0.13.2. Volcano plots were created using the R package ggplot2. Statistical significance was set to *p* < 0.05.

## 5. Results

### 5.1. Patient Characteristics

Patient baseline characteristics are summarized in Table 1. A total of 309 patients were included in the study, of whom 135, 66, and 108 were from QL, QLQD, and TCIA, respectively. A total of 243 patients from QL and TCIA were randomized into the training (*n* = 170, 69.9%) and internal validation (*n* = 73, 30.1%) cohorts, whereas QLQD was used as the independent cohort for external validation. Baseline data showed no statistically significant differences in terms of sex, age, lesion location, or pathological stage among the three cohorts, indicating their comparability. PU occurred in 52, 19, and 27 patients in the training, internal validation, and external validation cohorts, respectively.

### 5.2. PU Prediction Potential of the Tumor-Adjacent Region

**Figure 2:**
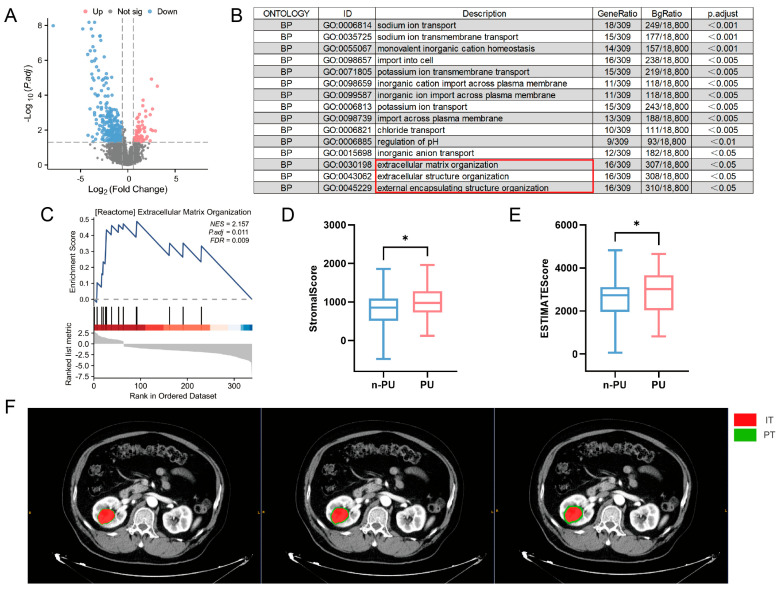
Bioinformatics analysis between non-PU and PU groups of patients with clear cell renal cell carcinoma. (**A**) Differential gene expression analysis of non-PU and PU samples. (**B**) Gene ontology enrichment analysis of differential genes. (**C**) Gene set enrichment analysis of differential genes. (**D**,**E**) ESTIMATE analysis indicated significant differences in the stromal score and ESTIMATE score between non-PU and PU groups. (**F**) Calibration schematic diagram of intratumoral (IT), 1-mm peritumoral (PT1), 2-mm peritumoral (PT2), and 3-mm peritumoral (PT3) regions, with red representing IT and green representing PT. * *p* < 0.05.

To explore imaging regions with the potential to predict PU, we analyzed the expression of differential genes for 108 patients from TCIA (Figure 2A). The differential genes were significantly enriched in extracellular matrix-related pathways (Figure 2B,C). To verify these results, we used the ESTIMATE algorithm to evaluate the tumor stroma of each sample. Patients with PU had higher tumor stromal contents (Figure 2D,E). Tumor stroma is an important component of the tumor microenvironment usually located in the tumor-adjacent region during imaging. Therefore, we expanded the previously outlined tumor region to obtain tumor-adjacent regions located within 1 mm (PT1), 2 mm (PT2), and 3 mm (PT3) of the tumor edge (Figure 2F) for further analysis. It should be clearly noted that the above transcriptomic and bioinformatics analysis in this study serves as exploratory biological justification for our subsequent peritumoral radiomics analysis, rather than establishing a direct quantitative linkage between individual imaging features and gene expression patterns. This analysis was performed to identify the critical biological role of the tumor-perirenal fat interface in the pathological upstaging of ccRCC, thereby providing a theoretical rationale for our inclusion of peritumoral regions in the radiomics model construction.

### 5.3. Extraction and Screening of Radiomic Features in Tumor and Surrounding Tissues

**Figure 3:**
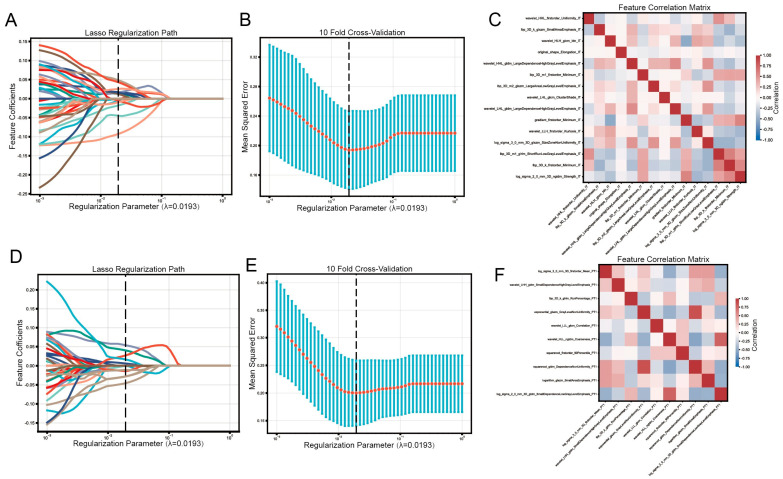
Screening of radiomic features in IT and PT1 regions. LASSO analysis (**A**,**B**) and Pearson’s correlation analysis (**C**) of radiomic features in IT. LASSO analysis (**D**,**E**) and Pearson’s correlation analysis (**F**) of radiomic features in PT1. Different colored lines represent distinct radiomic features in Figure 4A,D.

We extracted 1834 radiomic features from the renal cortex CT images and 5502 radiomic features from the surrounding tissue. After the five feature screening steps, we retained 15 radiomic features from the tumor, 10 radiomic features from PT1, 14 radiomic features from PT2, and 11 radiomic features from PT3 (Table 2). Lasso and Pearson’s correlation analyses showed that all selected features were fully regularized, with correlation coefficients < 0.7 for all feature pairs (Figure 3 and Figure S2), indicating low multicollinearity.

**Figure 4:**
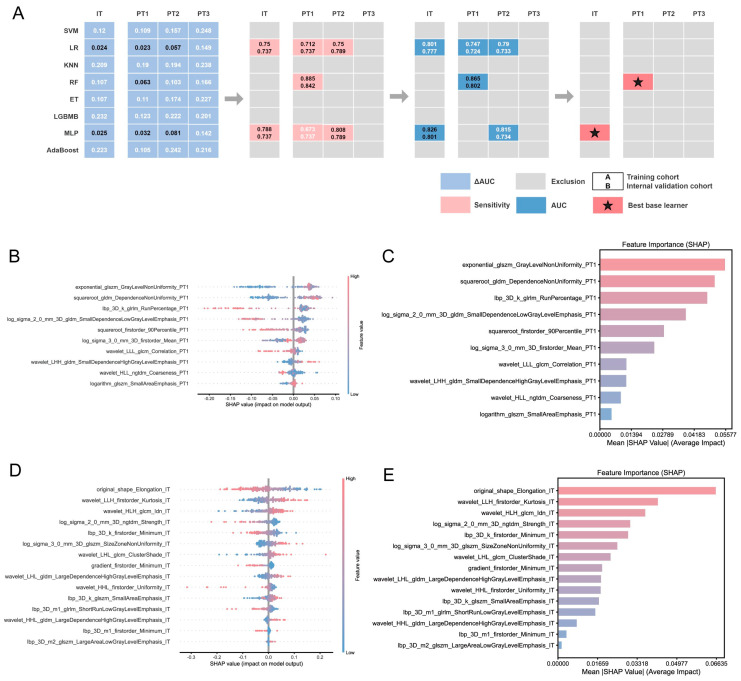
Selection of the optimal base learner. (**A**) Screening process of eight IT base learners and 24 PT1 base learners. The numbers in the upper and lower rows denote the Base Model Performance Metrics of the training cohort and internal validation cohorts, respectively. SHAP analysis swarm plot (**B**) and variable importance plot (**C**) of the optimal PT base learner (PT1-RF). SHAP analysis swarm plot (**D**) and variable importance plot (**E**) of the optimal IT base learner (IT-MLP).

### 5.4. Selection of the Optimal Base Learner

We developed eight machine learning models for internal tumors and 24 for external tumors; the performance of all 32 models is summarized in Tables S3–S6. IT-LR, IT-MLP, PT1-LR, PT1-RF, PT1-MLP, PT2-LR, and PT2-MLP performed consistently in the training and internal validation cohorts (ΔAUC < 0.1), indicating good generalization ability, which is crucial for clinical applications. As the incidence of PU in ccRCC is low, the developed model should also exhibit high sensitivity. IT-LR, IT-MLP, PT1-LR, PT1-RF, PT1-MLP, and PT2-LR showed good sensitivity (sensitivity > 0.7) in both cohorts. By further combining the AUC of the base learners in the training and internal validation sets, we ultimately selected IT-MLP and PT1-RF as the best base learners for tumor and non-tumor regions, respectively (Figure 4A). SHAP analysis showed that exponential_glszm_GrayLevelNonUniformity_PT1 was the most important feature for PT1-RF (Figure 4B,C), whereas original_shape_Elongation_IT was the most important feature for IT-MLP (Figure 4D,E).

To further elucidate the biological basis and clinical interpretability of our model beyond feature importance ranking, we contextualized the top predictive features identified by SHAP analysis with our transcriptomic findings. The most impactful feature for the PT1-RF base learner, exponential_glszm_GrayLevelNonUniformity_PT1, quantifies the heterogeneity of gray level distribution in the 1-mm peritumoral region. Higher values of this feature indicate greater disorganization of the peritumoral tissue microstructure, which aligns directly with our bioinformatics analysis showing significant enrichment of extracellular matrix remodeling pathways and higher stromal scores in the PU group. This texture metric captures microscopic tumor infiltration and stromal proliferation in the peritumoral invasive front, the core biological event driving pathological upstaging that is undetectable via conventional visual CT assessment. For the IT-MLP base learner, the top feature original_shape_Elongation_IT reflects the three-dimensional morphological regularity of the primary tumor. Lower elongation values correspond to a more irregular, infiltrative tumor shape, a well-established radiographic marker of aggressive ccRCC behavior widely used in subjective preoperative evaluation. Our model translates this qualitative visual assessment into a quantitative, reproducible metric. Collectively, these key radiomic features not only underpin the predictive performance of CRIPEM, but also provide surgeons with quantifiable preoperative imaging markers to stratify PU risk, inform the trade-off between partial and radical nephrectomy, and optimize individualized surgical decision-making.

### 5.5. Development and Validation of CRIPEM

**Figure 5:**
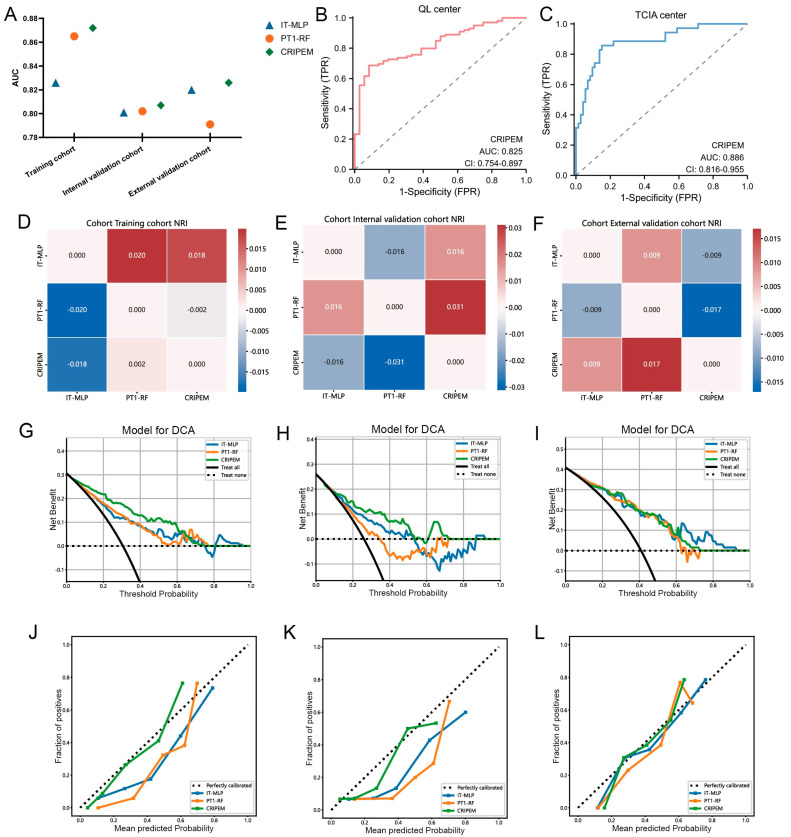
Development and validation of CRIPEM. (**A**) AUC values for the individual base learners IT-MLP and PT1-RF and the ensemble learning model CRIPEM in the training, internal validation, and external validation cohorts. (**B**,**C**) Subgroup analysis showed good performance for CRIPEM in QL and TCIA. (**D**–**F**) Net reclassification improvement analysis, (**G**–**I**) DCA analysis, and (**J**–**L**) calibration curve of CRIPEM in the training cohort (**D**,**G**,**J**), internal validation cohort (**E**,**H**,**K**), and external validation cohort (**F**,**I**,**L**).

We developed the CRIPEM integrated learning model by fusing IT-MLP and PT1-RF. CRIPEM outperformed the two base learners in terms of overall performance (AUC = 0.872) and maintained excellent predictive performance in both the internal and external validation sets (AUC = 0.807 and 0.826, respectively; Figure 5A, Table S7). Subgroup analysis confirmed that CRIPEM performed consistently in both QL and TCIA (Figure 5B,C). According to net reclassification improvement analysis, CRIPEM showed better predictive performance than the individual base learners in all three cohorts (Figure 5D–F). Decision curve analysis revealed that CRIPEM can provide a more substantial clinical net benefit within a wide range of threshold probabilities (Figure 5G–I). Calibration curves indicated that the predicted probability in training, internal validation, and external validation sets was highly consistent with the observed results (Figure 5J–L). Overall, our integrated learning strategy significantly improved model performance, and CRIPEM demonstrated high accuracy, stability, and clinical value in predicting PU.

**Figure 6:**
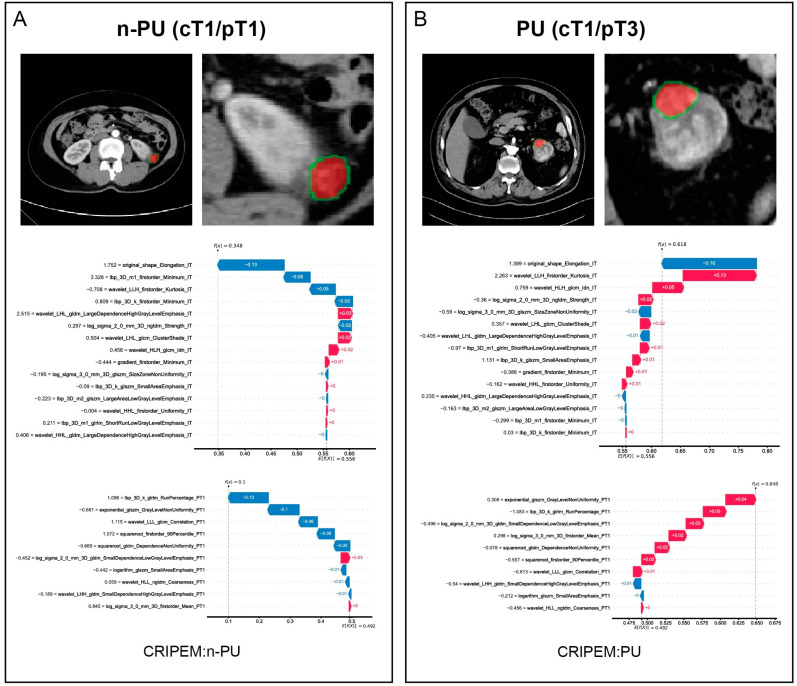
SHAP analysis of CRIPEM in predicting typical patients. Patient (**A**): preoperative diagnosis of cT1, postoperative pathology confirmed pT1 with no PU. Force plots show that IT-MLP, PT1-RF, and CRIPEM predicted the patient as non-PU, which was consistent with the actual situation. Patient (**B**): preoperative diagnosis of cT1, postoperative pathology confirmed pT3 with PU. Force plots show that all three models predicted the patient as PU, which was consistent with the actual situation.

To further demonstrate the clinical applicability of this model, we performed two individualized predictions. Patient 1 (Figure 6A) was diagnosed with cT1 preoperatively and pT1 postoperatively with no PU. According to force plots, the IT-MLP and PT1-RF base learning models and the integrated CRIPEM classed this patient as non-PU, consistent with the actual situation. Patient 2 (Figure 6B) was diagnosed as cT1 preoperatively and pT1 postoperatively, then confirmed as pT3 and pathologically upstaged. According to force plots, both base learners and CRIPEM classed the patient as PU, consistent with the actual situation.

## 6. Discussion

The management of localized RCC, a heterogeneous malignancy with increasing incidence, relies primarily on surgical resection [18]. However, the critical choice between nephron-sparing partial nephrectomy and radical nephrectomy depends on accurate stage assessment, which is hindered by a current reliance on conventional imaging methods [19,20,21]. Specifically, a key factor complicating surgical decisions is PU, in which postoperative histology reveals a higher tumor stage than clinically predicted [22]. Variability in PU behavior underscores the need for accurate preoperative prognostic tools to better stratify patient risks.

In this study, we systematically developed and evaluated the CRIPEM, which predicts PU in ccRCC by integrating radiomics with tumor subregion cluster analysis. Evaluation of the transcriptomic landscape, including differential expression and pathway enrichment, revealed that peritumoral areas provide critical information for PU prediction. From a multicenter cohort, we initially extracted 7336 radiomic features (1834 tumors, 5502 peritumoral). Following a rigorous selection process involving LASSO regression, z-score normalization, and Pearson’s correlation analysis, 50 robust features were retained. Finally, we trained and compared eight intratumoral and 24 peritumoral machine learning models, ultimately selecting two optimal base learners for tumor and non-tumor regions. Evaluation of model performance demonstrated that CRIPEM achieved superior predictive performance to the individual base learners, highlighting its potential applications for tailoring surgical strategies.

Our study addressed a key limitation of current clinical practice by providing an objective quantitative model for predicting PU. Although partial and radical nephrectomy are standard options for localized RCC, the optimal choice for patients ultimately upstaged to pT3a remains controversial. Although recent studies have reported comparable operative metrics and survival outcomes between partial and radical nephrectomy in broad cohorts, specific evidence suggests that partial nephrectomy may be associated with inferior oncological outcomes in patients with PU [23]. By enabling a more accurate preoperative risk stratification of PU, our predictive model can inform this critical surgical decision point, helping to identify patients that might benefit from a more aggressive initial resection versus or for whom partial nephrectomy remains more appropriate. Previous predictive models based on multiple parameters are limited by their complex and indirect nature [24], whereas our proposed model exploits the bioinformatics finding that radiomic features capturing the spatial relationship at the tumor–kidney interface contain critical predictive information [25]. CRIPEM was explicitly designed to quantify this interface, thereby providing a more accurate and objective calculation of PU probability. Notably, our CRIPEM yielded AUC values of 0.807 and 0.826 in the internal and external validation cohorts, respectively, which were markedly higher than the 0.70 AUC of the clinical model consisting of R.E.N.A.L. score, LMR, AGR, and PNI in a prior study [26]. Even when that clinical model was improved with LDL-C and CRI-I to achieve an AUC of 0.86 in a subgroup with complete lipid data, our radiomics-based CRIPEM provided comparable discriminative performance without additional serum lipid tests, indicating its distinct clinical added value.

Beyond direct surgical decision-making, the AI-based predictive framework developed in this study also holds promising translational potential for integration with artificial intelligence-assisted surgical robotic systems in minimally invasive nephrectomy. Artificial intelligence has already been applied to surgical robotic platforms in the minimally invasive treatment of other neoplastic diseases, with excellent results in optimizing preoperative planning, intraoperative precision manipulation, and perioperative risk stratification [27]. The PU risk probability output by CRIPEM can be used as a key preoperative stratification indicator for robotic minimally invasive surgery: for patients preoperatively identified as high PU risk by our model, the surgical team can preoperatively adjust the robotic surgical plan, prioritize radical nephrectomy with wider resection margins, and optimize intraoperative robotic manipulation strategies to ensure oncological safety; for low PU risk patients, the robotic platform can be better leveraged to perform nephron-sparing partial nephrectomy with maximal preservation of renal function. This integration of preoperative AI risk prediction and intraoperative AI-assisted robotic surgery represents a promising direction for precision nephron-sparing surgery in cT1 ccRCC.

Our study had some limitations. First, CRIPEM was developed and validated primarily for ccRCC, the most common RCC subtype; therefore, its performance in non-clear cell variants requires further investigation. Second, we collected data from only three centers; thus, the sample size remained moderate. Prospective validation in larger randomized cohorts is warranted to establish generalizability and assess the impact on long-term outcomes. Third, the proposed model currently relies on preoperative CT images. Therefore, integrating multimodal data, such as magnetic resonance imaging, electrochemical therapy, and clinical biomarkers, could further enhance its predictive efficiency. Fourth, the sample size of our external validation cohort is relatively modest (*n* = 66), which may limit the statistical power to fully validate the generalizability of CRIPEM. Although we confirmed the baseline comparability between the external and training cohorts and implemented preprocessing steps to mitigate imaging-related domain shifts, prospective multicenter validation with larger sample sizes across more clinical centers is warranted to further confirm the robustness and generalizability of our model in real-world clinical settings.

In conclusion, we present a multicenter retrospective radiomics-based predictive model (CRIPEM) for assessing the risk of PU in ccRCC, which can be used to improve surgical decision-making and clinical management.

## Figures and Tables

**Table 1:** Baseline data of patients included in this study.

Characteristics	Training Cohort	Internal Validation Cohort	External Validation Cohort
n	170	73	66
Gender, n (%)			
Male	118 (38.2%)	50 (16.2%)	43 (13.9%)
Female	52 (16.8%)	23 (7.4%)	23 (7.4%)
Age, mean ± sd	58.776 ± 10.994	60.658 ± 12.789	59.152 ± 11.468
Laterality, n (%)			
Left	76 (24.6%)	33 (10.7%)	30 (9.7%)
Right	94 (30.4%)	40 (12.9%)	36 (11.7%)
Pathologic stage, n (%)			
Stage I/II	116 (37.5%)	51 (16.5%)	39 (12.6%)
Stage III/IV	54 (17.5%)	22 (7.1%)	27 (8.7%)
Postoperative pathological upgrading, n (%)			
n-PU	118 (38.2%)	54 (17.5%)	39 (12.6%)
PU	52 (16.8%)	19 (6.1%)	27 (8.7%)

**Table 2:** Feature coefficient plots of LASSO-screened radiomic features in IT and PT1.

Value	Feature
−0.004965	wavelet_HHL_firstorder_Uniformity_IT
0.012804	lbp_3D_k_glszm_SmallAreaEmphasis_IT
0.017324	wavelet_HLH_glcm_Idn_IT
−0.092831	original_shape_Elongation_IT
0.016075	wavelet_HHL_gldm_LargeDependenceHighGrayLevelEmphasis_IT
−0.017977	lbp_3D_m1_firstorder_Minimum_IT
0.012177	lbp_3D_m2_glszm_LargeAreaLowGrayLevelEmphasis_IT
0.048574	wavelet_LHL_glcm_ClusterShade_IT
0.043215	wavelet_LHL_gldm_LargeDependenceHighGrayLevelEmphasis_IT
−0.000801	gradient_firstorder_Minimum_IT
0.015814	wavelet_LLH_firstorder_Kurtosis_IT
0.025466	log_sigma_3_0_mm_3D_glszm_SizeZoneNonUniformity_IT
−0.044672	lbp_3D_m1_glrlm_ShortRunLowGrayLevelEmphasis_IT
−0.005105	lbp_3D_k_firstorder_Minimum_IT
−0.009746	log_sigma_2_0_mm_3D_ngtdm_Strength_IT
0.006119	log_sigma_3_0_mm_3D_firstorder_Mean_PT1
0.053938	wavelet_LHH_gldm_SmallDependenceHighGrayLevelEmphasis_PT1
−0.045813	lbp_3D_k_glrlm_RunPercentage_PT1
0.040165	exponential_glszm_GrayLevelNonUniformity_PT1
−0.027867	wavelet_LLL_glcm_Correlation_PT1
−0.014285	wavelet_HLL_ngtdm_Coarseness_PT1
−0.028308	squareroot_firstorder_90Percentile_PT1
0.027226	squareroot_gldm_DependenceNonUniformity_PT1
0.016682	logarithm_glszm_SmallAreaEmphasis_PT1
−0.010914	log_sigma_2_0_mm_3D_gldm_SmallDependenceLowGrayLevelEmphasis_PT1
0.007703	exponential_firstorder_Kurtosis_PT2
0.044501	exponential_glszm_GrayLevelNonUniformity_PT2
0.003677	log_sigma_3_0_mm_3D_firstorder_Mean_PT2
0.019734	square_glrlm_GrayLevelNonUniformity_PT2
0.030673	wavelet_HHL_firstorder_Skewness_PT2
−0.046158	wavelet_LLL_glcm_Correlation_PT2
−0.017311	log_sigma_2_0_mm_3D_ngtdm_Contrast_PT2
−0.011341	wavelet_HLL_gldm_SmallDependenceLowGrayLevelEmphasis_PT2
0.045926	lbp_3D_m1_glrlm_RunEntropy_PT2
−0.025238	exponential_gldm_DependenceNonUniformityNormalized_PT2
−0.000671	wavelet_HLL_ngtdm_Strength_PT2
−0.022547	squareroot_firstorder_90Percentile_PT2
0.045262	wavelet_LHH_gldm_HighGrayLevelEmphasis_PT2
0.004121	wavelet_LHL_glcm_Imc1_PT2
0.021682	exponential_glszm_GrayLevelNonUniformity_PT3
−0.041323	wavelet_HHH_glcm_ClusterShade_PT3
0.025541	lbp_3D_m2_glszm_LargeAreaLowGrayLevelEmphasis_PT3
0.003028	square_glszm_ZoneVariance_PT3
0.008566	lbp_3D_k_firstorder_Maximum_PT3
−0.041656	wavelet_LLL_glcm_Correlation_PT3
0.029856	lbp_3D_m2_glrlm_RunEntropy_PT3
0.028112	log_sigma_2_0_mm_3D_glcm_Idmn_PT3
−0.001543	exponential_gldm_DependenceNonUniformityNormalized_PT3
−0.003687	logarithm_firstorder_90Percentile_PT3
0.044919	wavelet_LLH_glcm_Imc1_PT3

## Data Availability

The data generated and analyzed in this study are available from the corresponding author upon reasonable request.

## Supplementary Materials

The following supporting information can be downloaded at: https://www.mdpi.com/article/10.3390/cancers18101558/s1, Figure S1: Flow diagram of the study enrollment patients; Figure S2: LASSO analysis and Pearson correlation analysis of radiomic features for PT2 and PT3; Table S1: QL CT scanning parameters used for center and QLQDG center; Table S2: Coefficient plots of radiomic features selected by LASSO for PT2 and PT3; Table S3: Performance of base learners based on intratumoral radiomics in the training cohort and internal validation cohort; Table S4: Performance of base learners based on peritumoral 1 mm radiomics in the training cohort and internal validation cohort; Table S5: Performance of base learners based on peritumoral 2 mm radiomics in the training cohort and internal validation cohort; Table S6: Performance of base learners based on peritumoral 3 mm radiomics in the training cohort and internal validation cohort; Table S7: Performance of CRIPEM.
