# Supplementary material for: A CT-Based Radiomics Ensemble Model (CRIPEM) for Preoperative Prediction of Pathological Upstaging in Clear Cell Renal Cell Carcinoma
Source: Cancers (Basel). 2026 May 11;18(10):1558. doi: 10.3390/cancers18101558 (PMC13204160; doi:10.3390/cancers18101558)
Supplement: Supplementary file 1 [file cancers-18-01558-s001.zip › cancers-4276724-supplementary.pdf]

Supplementary Material

I Inclusion and Exclusion Criteria (English Translation)

Inclusion Criteria

1. Postoperative pathology confirms clear cell renal cell carcinoma;
2. Preoperative CT examination was performed, with CT image slice thickness ≤ 1mm, no motion artifacts/metal artifacts, good enhancement effect in the renal cortical phase, which can meet the requirements for radiomic feature extraction;
3. Complete clinicopathological data are available, including age, gender, lesion location, tumor size, Fuhrman grade, pathological stage, LMR/PNI indicators, etc.;
4. Clinical stage is cT1 (maximum tumor diameter ≤ 7cm and confined to the renal parenchyma).

Exclusion Criteria

1. Complicated with malignant tumors of other systems;
2. Preoperative anti-tumor treatments such as radiotherapy, chemotherapy, targeted therapy or immunotherapy were received;
3. CT images have severe artifacts, motion blur and other factors affecting image analysis;
4. Clinicopathological data are missing or incomplete.

II **Details of Radiomic Feature Extraction**

Before extracting radiomic features, linear interpolation was first used to resample image voxels to $1 \times 1 \times 1$ mm³, and the window level (WL = 50) and window width (WW = 350) were uniformly set to ensure the consistency of images from different centers. Subsequently, based on the PyRadiomics toolkit (https://github.com/Radiomics/pyradiomics) and with reference to the Imaging Biomarker Standardization Initiative (IBSI) guidelines, intratumoral and peritumoral radiomic features were extracted from CT images. A total of 7336 features were extracted, including first-order statistics, shape features of intratumoral and peritumoral 1, 2, and 3mm regions of clear cell renal cell carcinoma, as well as texture features based on gray-level run length matrix (GLRLM), gray-level size zone matrix (GLSZM), gray-level co-occurrence matrix (GLCM), gray-level dependence matrix (GLDM), and neighboring gray-tone difference matrix (NGTDM). In addition, derived features were obtained through various image transformations, including Laplacian of Gaussian (LoG), Wavelet, LBP3D, Exponential, Square, Square Root, Logarithm, and Gradient filtering.

III Development Method of Ensemble Learning Model

To determine the optimal intratumoral and peritumoral base learners, we used 8 machine learning algorithms (including Support Vector Machine (SVM), Logistic Regression (LR), K-Nearest Neighbors (KNN), Random Forest (RF), Extra Trees (ET), Light Gradient Boosting Machine (LightGBM), Multilayer Perceptron (MLP), and Adaptive Boosting (AdaBoost)) to develop base learners. The optimal base learner was selected by comparing the performance of each base learner on the training set and internal validation set. The screening criteria are as follows: (1) The difference in AUC of the base learner between the training cohort and the internal validation cohort is < 0.1; (2) The sensitivity of the base learner in both the training cohort and the internal validation cohort

is > 0.7; (3) On the premise of meeting the above criteria, the base learner with the highest AUC in both the training cohort and the internal validation cohort is preferentially selected as the optimal base learner. Subsequently, the prediction results of the optimal base learners for IT and PT1 were used as new prediction features, and a logistic regression was used to construct an ensemble learning model, which is called the CT-based ccRCC Intratumoral and Peritumoral radiomic Ensemble learning Model (CRIPEM).

Figure S1. Flow diagram of the study enrollment patients.

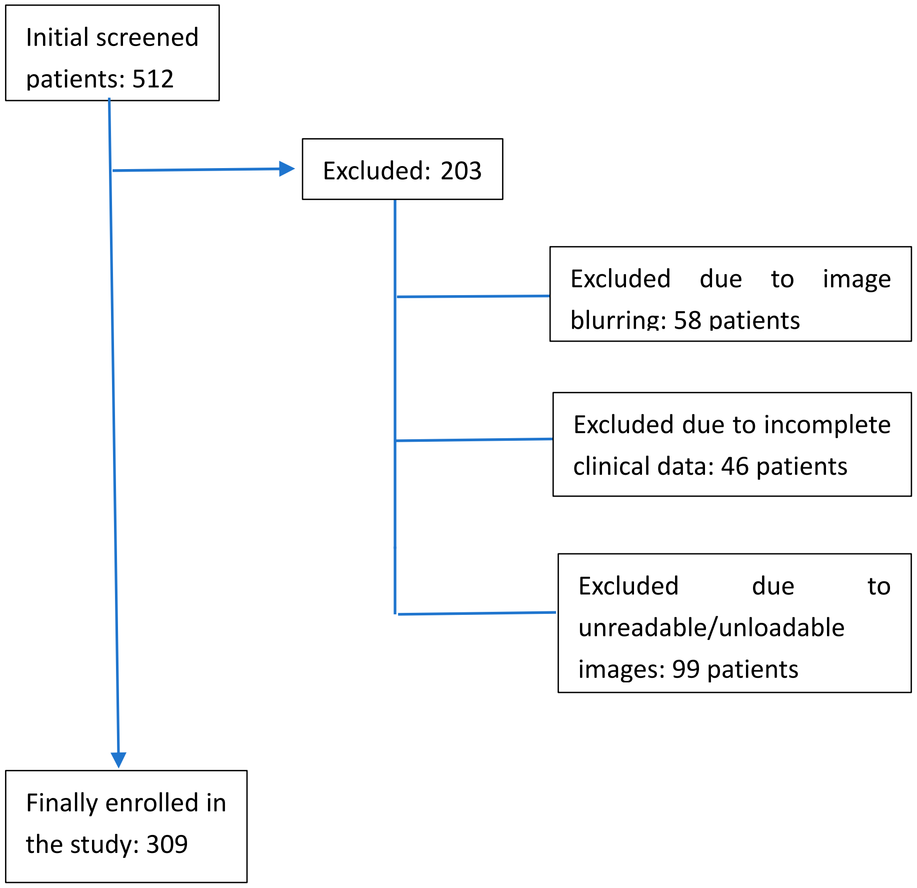

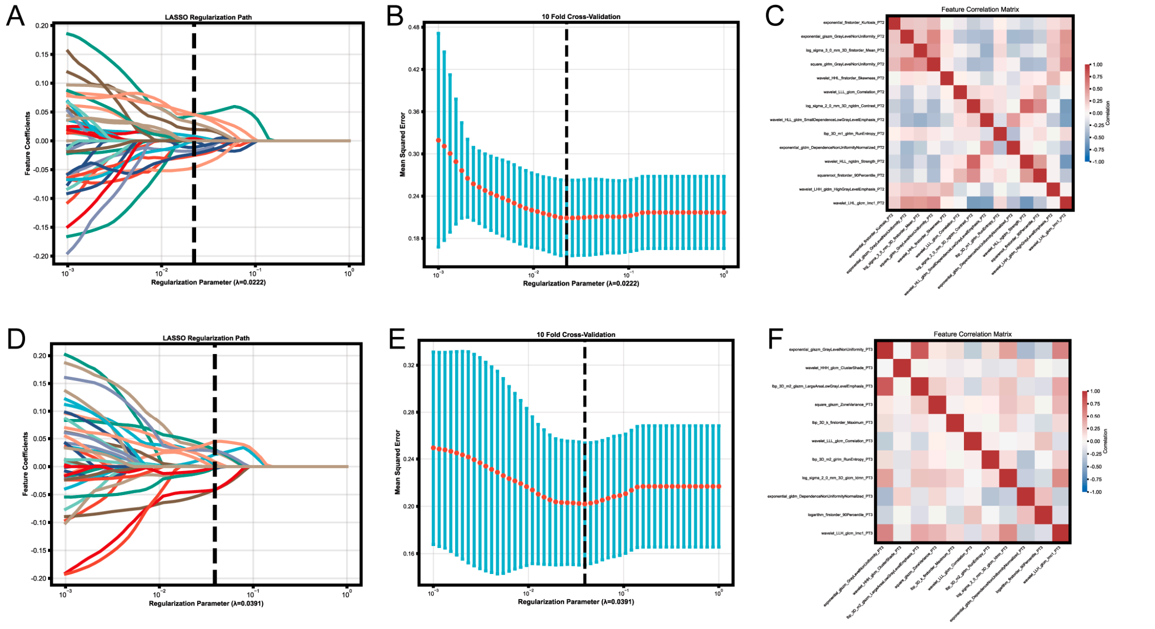

Figure S2. LASSO analysis and Pearson correlation analysis of radiomic features for PT2 and PT3.LASSO analysis (A, B) and Pearson correlation analysis (C) of radiomic features for PT2.LASSO analysis (D, E) and correlation analysis (F) of radiomic features for PT3.

Table S1. QL CT scanning parameters used for center and QLQDG center.

Scanner	CT tube voltage	CT tube current	CT detector collimation	Contrast agent concentration	Reconstruction Matrix
GE Revolution Apex	100-120Kv	Automatic	256×0.625 mm	300 mgl/ml	512 * 512
SOMATOM Force	Automatic	Automatic	96×2×0.625 mm	300 mgl/ml	512 * 512
SOMATOM Drive	Automatic	Automatic	64×2×0.625 mm	300 mgl/ml	512 * 512

kV= kilovolt; mA=milliampere; mm= milietre.

Table S2. Coefficient plots of radiomic features selected by LASSO for PT2 and PT3.

Category	Coefficient	Radiomics feature
PT2	0.007703	exponential_firstorder_Kurtosis_PT2
	0.044501	exponential_glszm_GrayLevelNonUniformity_PT2
	0.003677	log_sigma_3_0_mm_3D_firstorder_Mean_PT2
	0.019734	square_glrlm_GrayLevelNonUniformity_PT2
	0.030673	wavelet_HHL_firstorder_Skewness_PT2
	-0.046158	wavelet_LLL_glcm_Correlation_PT2
	-0.017311	log_sigma_2_0_mm_3D_ngtdm_Contrast_PT2
	-0.011341	wavelet_HLL_gldm_SmallDependenceLowGrayLevelEmphasis_PT2
	0.045926	lbp_3D_m1_glrlm_RunEntropy_PT2
	-0.025238	exponential_gldm_DependenceNonUniformityNormalized_PT2
	-0.000671	wavelet_HLL_ngtdm_Strength_PT2
	-0.022547	squareroot_firstorder_90Percentile_PT2
	0.045262	wavelet_LHH_gldm_HighGrayLevelEmphasis_PT2
	0.004121	wavelet_LHL_glcm_Imc1_PT2

	0.021682	exponential_glszm_GrayLevelNonUniformity_PT3
	-0.041323	wavelet_HHH_glcm_ClusterShade_PT3
	0.025541	lbp_3D_m2_glszm_LargeAreaLowGrayLevelEmphasis_PT3
	0.003028	square_glszm_ZoneVariance_PT3
	0.008566	lbp_3D_k_firstorder_Maximum_PT3
PT3	-0.041656	wavelet_LLL_glcm_Correlation_PT3
	0.029856	lbp_3D_m2_glrlm_RunEntropy_PT3
	0.028112	log_sigma_2_0_mm_3D_glcm_Idmn_PT3
	-0.001543	exponential_gldm_DependenceNonUniformityNormalized_PT3
	-0.003687	logarithm_firstorder_90Percentile_PT3
	0.044919	wavelet_LLH_glcm_Imc1_PT3

Table S3. Performance of base learners based on intratumoral radiomics in the training cohort and internal validation cohort**.**

Base learner	Accuracy	AUC	95% CI	Sensitivity	Specificity	PPV	NPV	F1	Label
IT-SVM	0.812	0.877	0.8220 - 0.9319	0.846	0.797	0.647	0.922	0.733	Training cohort
IT-SVM	0.699	0.757	0.6328 - 0.8818	0.632	0.722	0.444	0.848	0.522	Internal validation cohort
IT-LR	0.706	0.801	0.7290 - 0.8740	0.75	0.686	0.513	0.862	0.609	Training cohort
IT-LR	0.685	0.777	0.6547 - 0.8989	0.737	0.667	0.437	0.878	0.549	Internal validation cohort
IT-KNN	0.729	0.879	0.8290 - 0.9285	0.923	0.644	0.533	0.95	0.676	Training cohort
IT-KNN	0.534	0.67	0.5331 - 0.8060	0.789	0.444	0.333	0.857	0.469	Internal validation cohort
IT-RF	0.765	0.883	0.8309 - 0.9354	0.865	0.72	0.577	0.924	0.692	Training cohort
IT-RF	0.767	0.776	0.6677 - 0.8840	0.632	0.815	0.545	0.863	0.585	Internal validation cohort
IT-ET	0.788	0.909	0.8644 - 0.9544	0.904	0.737	0.603	0.946	0.723	Training cohort
IT-ET	0.781	0.802	0.6857 - 0.9186	0.842	0.759	0.552	0.932	0.667	Internal validation cohort
IT-LGBMB	0.853	0.933	0.8958 - 0.9708	0.846	0.856	0.721	0.927	0.779	Training cohort
IT-LGBMB	0.658	0.701	0.5769 - 0.8247	0.421	0.741	0.364	0.784	0.39	Internal validation cohort
IT-MLP	0.765	0.826	0.7584 - 0.8939	0.788	0.754	0.586	0.89	0.672	Training cohort
IT-MLP	0.753	0.801	0.6871 - 0.9153	0.737	0.759	0.519	0.891	0.609	Internal validation cohort
IT-AdaBoost	0.812	0.884	0.8371 - 0.9317	0.865	0.788	0.643	0.93	0.738	Training cohort
IT-AdaBoost	0.658	0.661	0.5277 - 0.7949	0.368	0.759	0.35	0.774	0.359	Internal validation cohort

Table S4. Performance of base learners based on peritumoral 1 mm radiomics in the training cohort and internal validation cohort.

Base learner	Accuracy	AUC	95% CI	Sensitivity	Specificity	PPV	NPV	F1	Label
PT1-SVM	0.712	0.817	0.7546 - 0.8793	0.769	0.686	0.519	0.871	0.62	Training cohort
PT1-SVM	0.603	0.708	0.5642 - 0.8510	0.737	0.556	0.368	0.857	0.491	Internal validation cohort
PT1-LR	0.694	0.747	0.6731 - 0.8201	0.712	0.686	0.5	0.844	0.587	Training cohort
PT1-LR	0.726	0.724	0.5935 - 0.8548	0.737	0.722	0.483	0.886	0.583	Internal validation cohort
PT1-KNN	0.729	0.88	0.8290 - 0.9319	0.885	0.661	0.535	0.929	0.667	Training cohort
PT1-KNN	0.63	0.69	0.5350 - 0.8441	0.737	0.593	0.389	0.865	0.509	Internal validation cohort
PT1-RF	0.729	0.865	0.8111 - 0.9187	0.885	0.661	0.535	0.929	0.667	Training cohort
PT1-RF	0.671	0.802	0.6802 - 0.9241	0.842	0.611	0.432	0.917	0.571	Internal validation cohort
PT1-ET	0.741	0.889	0.8411 - 0.9366	0.904	0.669	0.547	0.94	0.681	Training cohort
PT1-ET	0.644	0.779	0.6563 - 0.9012	0.789	0.593	0.405	0.889	0.536	Internal validation cohort
PT1-LGBMB	0.8	0.912	0.8688 - 0.9558	0.846	0.78	0.629	0.92	0.721	Training cohort
PT1-LGBMB	0.712	0.789	0.6724 - 0.9065	0.789	0.685	0.469	0.902	0.588	Internal validation cohort
PT1-MLP	0.676	0.783	0.7142 - 0.8519	0.673	0.678	0.479	0.825	0.56	Training cohort
PT1-MLP	0.699	0.751	0.6212 - 0.8818	0.737	0.685	0.452	0.881	0.56	Internal validation cohort
PT1-AdaBoost	0.741	0.838	0.7802 - 0.8967	0.808	0.712	0.553	0.894	0.656	Training cohort
PT1-AdaBoost	0.685	0.733	0.6046 - 0.8613	0.737	0.667	0.437	0.878	0.549	Internal validation cohort

Table S5. Performance of base learners based on peritumoral 2 mm radiomics in the training cohort and internal validation cohort**.**

Base learner	Accuracy	AUC	95% CI	Sensitivity	Specificity	PPV	NPV	F1	Label
PT2-SVM	0.806	0.902	0.8550 - 0.9481	0.808	0.805	0.646	0.905	0.718	Training cohort
PT2-SVM	0.74	0.745	0.6090 - 0.8803	0.684	0.759	0.5	0.872	0.578	Internal validation cohort
PT2-LR	0.7	0.79	0.7181 - 0.8615	0.75	0.678	0.506	0.86	0.605	Training cohort
PT2-LR	0.671	0.733	0.6059 - 0.8599	0.789	0.63	0.429	0.895	0.556	Internal validation cohort
PT2-KNN	0.765	0.896	0.8495 - 0.9416	0.885	0.712	0.575	0.933	0.697	Training cohort
PT2-KNN	0.575	0.702	0.5596 - 0.8448	0.684	0.537	0.342	0.829	0.456	Internal validation cohort
PT2-RF	0.776	0.901	0.8559 - 0.9466	0.827	0.754	0.597	0.908	0.694	Training cohort
PT2-RF	0.712	0.798	0.6647 - 0.9318	0.842	0.667	0.471	0.923	0.604	Internal validation cohort

PT2-ET	0.759	0.93	0.8910 - 0.9688	0.904	0.695	0.566	0.943	0.696	Training cohort
PT2-ET	0.685	0.756	0.6265 - 0.8862	0.789	0.648	0.441	0.897	0.566	Internal validation cohort
PT2-LGBMB	0.876	0.942	0.9075 - 0.9775	0.865	0.881	0.763	0.937	0.811	Training cohort
PT2-LGBMB	0.712	0.72	0.5752 - 0.8654	0.684	0.722	0.464	0.867	0.553	Internal validation cohort
PT2-MLP	0.735	0.815	0.7470 - 0.8834	0.808	0.703	0.545	0.892	0.651	Training cohort
PT2-MLP	0.699	0.734	0.6023 - 0.8655	0.789	0.667	0.455	0.9	0.577	Internal validation cohort
PT2-AdaBoost	0.8	0.877	0.8235 - 0.9296	0.731	0.831	0.655	0.875	0.691	Training cohort
PT2-AdaBoost	0.63	0.635	0.4947 - 0.7763	0.579	0.648	0.367	0.814	0.449	Internal validation cohort

AdaBoost: Adaptive Boosting
AUC: Area Under the Receiver Operating Characteristic Curve
CI: Confidence Interval
ET: Extra Trees
F1: F1 Score
KNN: K-Nearest Neighbors
LGBMB: Light Gradient Boosting Machine
LR: Logistic Regression
MLP: Multilayer Perceptron
NPV: Negative Predictive Value
PPV: Positive Predictive Value
PT2: peritumoral 2 mm
RF: Random Forest
SVM: Support Vector Machine

Table S6. Performance of base learners based on peritumoral 3 mm radiomics in the training cohort and internal validation cohort**.**

Base learner	Accuracy	AUC	95% CI	Sensitivity	Specificity	PPV	NPV	F1	Label
PT3-SVM	0.824	0.924	0.8804 - 0.9686	0.904	0.788	0.653	0.949	0.758	Training cohort
PT3-SVM	0.644	0.676	0.5464 - 0.8064	0.579	0.667	0.379	0.818	0.458	Internal validation cohort
PT3-LR	0.706	0.799	0.7284 - 0.8687	0.75	0.686	0.513	0.862	0.609	Training cohort
PT3-LR	0.616	0.65	0.5154 - 0.7848	0.632	0.611	0.364	0.825	0.462	Internal validation cohort
PT3-KNN	0.788	0.905	0.8593 - 0.9505	0.904	0.737	0.603	0.946	0.723	Training cohort
PT3-KNN	0.562	0.667	0.5338 - 0.8005	0.789	0.481	0.349	0.867	0.484	Internal validation cohort
PT3-RF	0.794	0.908	0.8608 - 0.9544	0.904	0.746	0.61	0.946	0.729	Training cohort
PT3-RF	0.699	0.742	0.6058 - 0.8776	0.737	0.685	0.452	0.881	0.56	Internal validation cohort

PT3-ET	0.812	0.941	0.9075 - 0.9755	0.942	0.754	0.628	0.967	0.754	Training cohort
PT3-ET	0.658	0.714	0.5712 - 0.8577	0.684	0.648	0.406	0.854	0.51	Internal validation cohort
PT3-LGBMB	0.865	0.941	0.9046 - 0.9783	0.846	0.873	0.746	0.928	0.793	Training cohort
PT3-LGBMB	0.63	0.74	0.6018 - 0.8777	0.737	0.593	0.389	0.865	0.509	Internal validation cohort
PT3-MLP	0.741	0.85	0.7899 - 0.9096	0.788	0.72	0.554	0.885	0.651	Training cohort
PT3-MLP	0.658	0.708	0.5723 - 0.8429	0.632	0.667	0.4	0.837	0.49	Internal validation cohort
PT3-AdaBoost	0.794	0.883	0.8339 - 0.9312	0.808	0.788	0.627	0.903	0.706	Training cohort
PT3-AdaBoost	0.616	0.667	0.5151 - 0.8192	0.789	0.556	0.385	0.882	0.517	Internal validation cohort

Table S7. Performance of CRIPEM.

Accuracy	AUC	95% CI	Sensitivity	Specificity	PPV	NPV	F1	Label
0.753	0.872	0.8195 - 0.9250	0.865	0.703	0.562	0.922	0.682	Training cohort
0.753	0.807	0.6879 - 0.9262	0.842	0.722	0.516	0.929	0.640	Internal validation cohort
0.727	0.826	0.7285 - 0.9239	0.963	0.564	0.605	0.957	0.743	External validation cohort

1. Evaluation of Tumor Microenvironment Stromal Score via the ESTIMATE Algorithm

To assess the differences in stromal components of the tumor microenvironment (TME) in clear cell renal cell carcinoma (ccRCC) samples, the ESTIMATE algorithm was adopted in this study for the quantitative analysis of stromal scores. Firstly, the expression matrices of samples stratified into the PU group and non-PU group were extracted from the TCGA dataset. The *Estimate* package in R software (version 4.2.0) was used to calculate the Stromal Score and Estimate Score of each sample with default parameters. These scores were derived from stromal cell-specific gene signatures of tumor tissues and calculated based on single-sample gene set enrichment analysis (ssGSEA); a higher score represents a higher abundance of stromal components in the tumor microenvironment. Afterwards, the Wilcoxon rank-sum test was used to compare the differences in stromal scores between the PU and non-PU groups, so as to clarify the changing characteristics of TME stromal components under the PU status.

2. Gene Set Enrichment Analysis (GSEA)

To explore the potential biological pathways and regulatory mechanisms mediated by differentially expressed genes between the PU group and n-PU group, gene set enrichment analysis (GSEA) was performed on the differential genes of the two subgroups. Firstly, the ranking metrics of all genes were calculated according to the $\log_2$ fold change ($\log_2$FC) and statistical significance of each gene obtained from differential expression analysis, and a pre-ranked gene list was generated. GSEA was

implemented using the *clusterProfiler* package in R software. The enrichment score (ES) was used to evaluate the distribution bias of gene sets in the ranked list, and the normalized enrichment score (NES) was applied to standardize gene different sizes.
